# Dynamics and Determinants of the Grain Yield Gap in Major Grain-Producing Areas: A Case Study in Hunan Province, China

**DOI:** 10.3390/foods11081122

**Published:** 2022-04-13

**Authors:** De Yu, Shougeng Hu, Luyi Tong, Cong Xia, Penglai Ran

**Affiliations:** 1 School of Public Administration, China University of Geosciences (CUG), Wuhan 430074, China; yydydd@cug.edu.cn (D.Y.); lytong@cug.edu.cn (L.T.); forestxc1998@cug.edu.cn (C.X.); ran@cug.edu.cn (P.R.); 2 Key Laboratory for Research on Rule of Law, Ministry of Natural Resources, Wuhan 430074, China

**Keywords:** yield gap, spatiotemporal variations, food security, determinants, machine learning

## Abstract

Understanding the dynamics of the grain yield gap (*YGAP*) and its causative factors is essential for optimizing the layout of grain production and addressing the food crisis, especially in countries with a huge population and less cultivated land, such as China. In the study, a spatial analysis- and machine learning-based framework for *YGAP* analysis was developed, taking Hunan Province, China, as an application. The results showed that the average *YGAP* in Hunan Province gradually narrowed from 1990 to 2018, and the *YGAPs* narrowed in 116 counties. Of which, 26 counties narrowed by more than 4 t ha^−1^, 58 counties narrowed from 2–4 t ha^−1^, and 32 counties narrowed within 2 t ha^−1^. Additionally, we found that the *GDP* per capita (*GDPPC*), sunshine hours (*SH*), per capita annual net income of farmers (*PCAI*), and rural electricity consumption (*REC*) play a key role in *YGAP* change, and the importance of human investment to the *YGAP* decreased, while socioeconomic environment became the dominant factor that influenced grain production. Comprehensively, the relatively great potential for grain yield growth was generated in sixty-four counties, which are mainly located in the northern, central, and southern Hunan. The findings suggest that it is necessary to consider the trends of economic development in rural areas and population migration in agricultural management. This work provides insights into yield gap dynamics and may contribute to sustainable agricultural management in Hunan Province, China, and other similar regions.

## 1. Introduction

Grain production capacity and food security have been wide concerns in the era of population boom, climate change, and environmental degradation [1,2,3,4,5]. Many scholars in developed countries have researched the sustainability and resilience of the food system since the 1990s, and it is concluded that a food system gathers all the elements (environment, people, inputs, processes, infrastructures, institutions, etc.) and activities that relate to the production, processing, distribution, preparation, and consumption of food and the outputs of these activities [6,7]. In 2015, zero hunger, which advocates sustainable agricultural development promotion to ensure global food security, was introduced as one of the UN’s sustainable development goals (SDGs) [8,9,10]. However, the food crisis remains and, to a certain extent, has been even worse since 2017 [11]. Affected by global socioeconomic and health events such as the COVID-19 pandemic, food supply chains, trade, and food environments are getting more vulnerable [12,13], and more than 155 million people are suffering from food insecurity [14]. Meanwhile, it is reported that more than 1.3 billion tons of food are thrown away along the entire food supply chain worldwide each year, especially in developed countries. Specifically, in developed countries (1.4 billion people), 670 million tons (Mt) of food is discarded, and less than 630 Mt is discarded in developing countries (6.2 billion people) [15,16,17]. In this context, initiatives to promote a resilient agricultural food system are unprecedentedly advocated [11]. Some scholars stated that the current food system is problematic because of alleged low productivity, especially in developing countries [13]. Indeed, there is a large gap in grain crops between developing and developed countries. For instance, the yield per unit of rice in China is more than 110 kg lower than that of Australia, the yield per unit of wheat is nearly 300 kg lower than that of New Zealand, the yield per unit of corn is only 60% of that of the United States, and the yield per unit of soybean is less than 60% of that in the United States [18]. Therefore, it is necessary to move beyond rehabilitating and increasing agricultural production to addressing the whole food system to link humanitarian assistance and longer-term support to sustainable livelihoods and resilience [6].

The Chinese government and scholars have made efforts to approach high-quality cultivated land and increase the sown area of grain to gain more food [19,20,21]. Policies such as cultivated land balance, land consolidation, and a high standard of basic cultivated land that aim to promote strict cultivated land protection have also been approached [22,23,24]. However, it remains hard work to address cultivated land degradation in both scale and grain production capacity under rapid urbanization and industrialization [25]. Low grain yields in major cereal crops, notably maize, rice, and wheat, have been generally generated in areas suffering from resource and environmental constraints [26,27,28]. Moreover, promoted by the policies addressing the decreasing birth rate, the population growth, and, as a result, the increase in food consumption will be largely increased in the next decades [29,30,31,32]. Food security is a major issue facing Chinese agriculture [20,32,33,34]. Comprehensively, increasing grain productivity without cultivated land expansion is critical to address these problems.

It is noteworthy that initiatives to increase the grain yield in regions that show a large yield gap (*YGAP*), namely the gap between potential yield (*YP*) and actual farm yield (*YAFM*), are advocated. The definition of *YGAP* was firstly made by De Datta (1981) and has been enriched during the past decades. Concepts related to *YGAP*, mainly including potential yield (*YP*), exploitable yield potential (*YEP*), potential farm yield (*YPFM*), and actual farm yield (*YAFM*), have been widely discussed [28,35,36,37,38]. Among these definitions, *YP* is the maximum yield that can be obtained under the climatic and soil conditions in a specific area. *YEP* reflects the maximum yield achievable in a test field under superior cultivation management practices. *YPFM* represents the maximum yield to be obtained at the current cultivation level by the farmer. In contrast, *YAFM* is the actual yield under the current farming practices [38,39,40]. Generally, production constraints are highly dependent on local management practices and agroecological location [41]. Of which, *YP* is mainly determined by natural conditions (e.g., light, temperature, water, soil, etc.) and genetic characteristics of crops. While the influence mechanism of *YAFM* is relatively complex, it is mainly decided by the land use conditions, the inputs of labor, technology, capital, etc. The inputs are conducive to improving the land use conditions, promoting cultivated land quality, and finally enhancing *YAFM*, while the willingness of farmers to input in agricultural activities is largely determined by the socioeconomic environment [38]. Hence, it can be seen that it is very difficult but crucial to explore the key factors that narrow the yield gap.

In recent years, studies on the *YGAP* have continuously deepened. The research object has expanded from major grain crops (rice, wheat, and corn) [42,43,44] to potatoes [45], sugarcane [46], rapeseed [47], quinoa [48], cassava [49,50], apples [51], bovine milk [52,53], and cowpeas [54]. While in terms of research method, field surveys, statistical methods, crop simulation models, and remote sensing technology have been integrated to fully utilize the advantages of each method in *YGAP* research, and insight has been derived through targeted case studies [55,56,57]. All of these have deepened the cognition of yield gap research. However, findings documented in existing literature are variable due to differences in research scales and methods. Specifically, most studies have been conducted at the field, provincial, regional, and national levels [58,59,60,61], ignoring the mesoscale such as the county level. Compared to the latter three scales, the field survey study can achieve more precise data and results. It is helpful to guide agricultural production in practice. Existing literature revealed that cultivation and management measures such as increasing fertilization, irrigation, and planting density have an important contribution to the increase in yield and then narrow the yield gap [62,63,64,65]. However, the field survey method is hard to promote within a large region because of the limits of finances and time. While using remote sensing and statistical data to research *YGAP* at macro-scales such as provincial, regional, and national, can quickly understand their trends and help strategic decisions making [66,67,68]. For instance, previous studies have revealed that the *YGAP* is particularly large in developing countries where smallholder farming dominates the agricultural landscape, especially in rainfed systems, suggesting that the increase in grain production in these regions is easier than in other places [53,69,70], but large-scale studies are difficult to guide grain production activities directly. Comprehensively, the *YGAP* study at the mesoscale is needed. Meanwhile, some studies reported that limiting factors to production are region-specific and depend on socio-economic and agro-ecological location; therefore, it is necessary to understand the primary causes of yield gaps to allow for more effective research and policy efforts aimed at improving grain production capacity [38,41].

Furthermore, considering many policies, such as spatial planning and agricultural industry planning, are implemented at the county level in China, and Deng et al. [20] reported that among the major rice-producing provinces, the greatest opportunity for yield improvement mainly occurs in Hunan, Heilongjiang, and Jiangxi in China. Meanwhile, to the best of our knowledge, a limited number of studies have examined the factors influencing the *YGAP*, especially from spatial and temporal perspectives at the county level. Hence, to develop a methodology framework from a geographical perspective for regional yield gap analysis and apply it to verify its effectiveness, Hunan Province, which is a major grain-producing base in China, was selected as a study area.

Specifically, we analyzed the spatiotemporal evolution characteristics of crop *YGAPs* and the corresponding determinants in Hunan Province based on various data (e.g., remote sensing data, meteorological data, and socioeconomic data) and methods (e.g., spatial analysis, spatial statistics, and random forest model). The specific objectives of this study are (a) to better understand the spatiotemporal evolution and clustering characteristics of the *YGAP* at the county level, (b) to determine the factors influencing the yield gap change (*YGC*), and (c) to identify areas with a high potential to narrow the *YGAP* and propose strategies to increase the grain yield. This study provides new insights into the application of *YGAP* research.

## 2. Materials and Methods

### 2.1. Study Area

**Figure 1:**
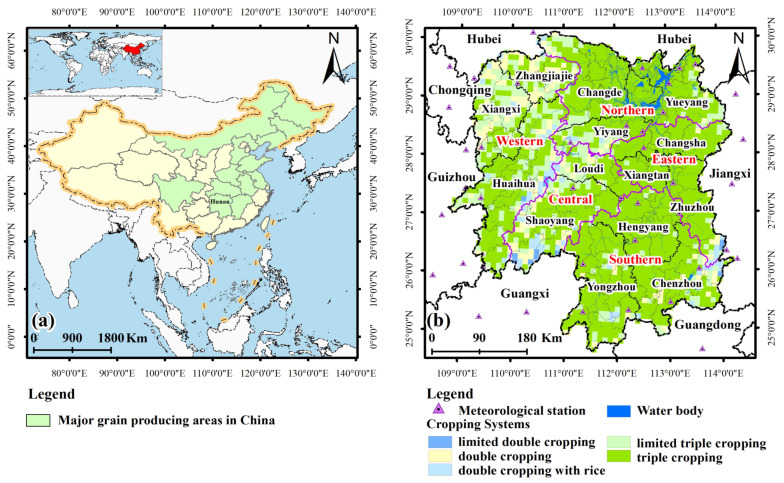
The location (**a**) and the cropping systems of Hunan Province (**b**).

**Figure 2:**
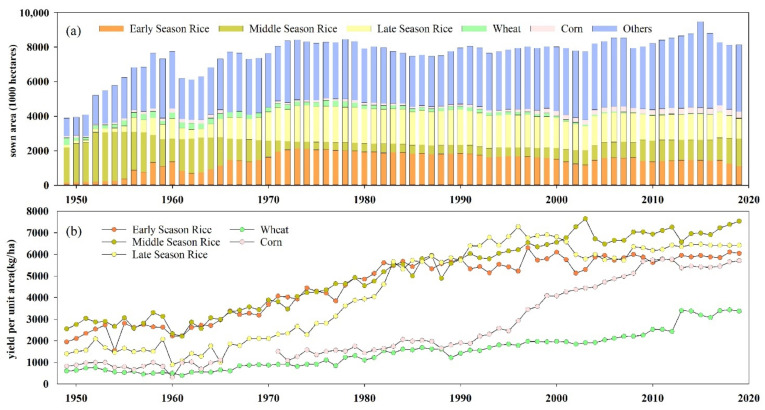
Sown area (**a**) and yield (**b**) of the crops planted in Hunan Province, China during 1949–2019.

There are thirteen provinces among the main grain-producing areas of China, including Liaoning, Hebei, Shandong, Jilin, Inner Mongolia, Jiangxi, Hunan, Sichuan, Henan, Hubei, Jiangsu, Anhui, and Heilongjiang (Figure 1a). Hunan, located in Central China and the middle reaches of the Yangtze River, between 108°47′–114°15′ E and 24°38′–30°08′ N, covers an area of 211,800 square kilometers and contains 14 municipalities and 122 counties or districts (Figure 1). It exhibits a humid continental subtropical monsoon climate with suitable agricultural production conditions (e.g., light, heat, and water resources), and is an important rice production base in China. The main grain cropping system in Hunan Province is double cropping with rice (early-season rice and late-season rice) (Figure 1b) [71]; however, an increasing number of farmers have preferred to plant rice in the middle of the two seasons in recent years (Figure 2a). In 2019, the area sown with rice accounted for approximately 90% of the total food crop planting area in Hunan (Figure 2a), and the rice yield reached 26.39 million tons, of which early-season rice accounted for 7.19 million tons, and late-season rice accounted for 8.1 million tons of the total rice yield. The yield per unit area of early-, middle- and late-season rice exceeded 6000 kg ha^−1^ in 2019 (Figure 2b). In 2020, the grain sown area in Hunan amounted to 4755 thousand hectares, and the total grain production reached 30.15 million tons, accounting for 4.51% of the grain yield in China.

### 2.2. Data Sources

Land use data with a spatial resolution of 30 m for 1990, 2000, 2010, and 2018 were provided by the RESDC, Chinese Academy of Sciences (http://www.resdc.cn, accessed on 1 December 2019). These data adopt a three-level classification system, which divides land into six primary categories, namely, cultivated land, woodland, grassland, water area, urban and rural construction land, and unused land, and 25 secondary categories, such as paddy fields and dryland areas. The detailed introduction can be seen in reference [72]. DEM data with a spatial resolution of 12.5 m were obtained from the Advanced Land Observing Satellite (ALOS), which is known in Japan as DAICHI and was developed by the Japan Aerospace Exploration Agency (JAXA, https://global.jaxa.jp, accessed on 13 March 2021). The rainfall, air temperature, sunshine hour, and solar radiation intensity data in 1990, 2000, 2010, and 2018 originated from the China Meteorological Data Network Service Centre (http://data.cma.cn, accessed on 13 February 2021) and National Tibetan Plateau Data Center (http://data.tpdc.ac.cn, accessed on 13 February 2021). Socioeconomic data (e.g., grain yield, human population, rural labor, etc.) were obtained from the Hunan Statistical Yearbook and Hunan Rural Statistical Yearbook of the corresponding year. Administrative district data were derived from basic national geodatabases.

### 2.3. Methods

#### 2.3.1. Framework for *YGAP* Analysis and Application

**Figure 3:**
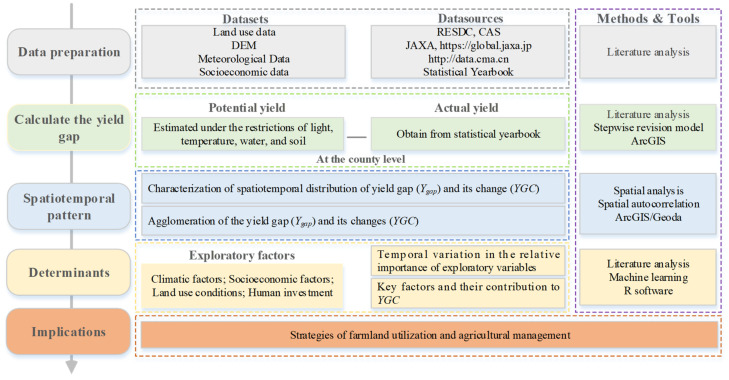
The overall methodological framework for yield gap analysis.

Theoretically, the yield gap is determined not only by natural conditions (e.g., the physical conditions of cultivated land and the climate) but also by human investments, such as irrigated infrastructure, technology, and capital [53,69,73,74,75,76]. The former aspect determines the grain production potential, and the latter aspect determines the actual grain outputs. When the exploitation level of the potential yield is low, the latter factor may be more important than the former factor. However, all the limiting factors vary over time and space, and the same investment does not produce the same benefits over places. Thus, to optimize the pattern and improve the efficiency of grain production, we need to understand some questions, such as how the yield gap changes, which factors determine the changes in the yield gap, and where and how we can close the *YGAP*. Therefore, a methodology and analysis framework was developed based on a geographic research perspective [77] in this study (Figure 3).

Firstly, the yield gap in each county in 1990, 2000, 2010, and 2018 was calculated. Then, we analyzed the characteristics of yield gap dynamics (e.g., the distribution, clustering, and evolution) with the methods of spatial analysis and spatial statistics. Finally, we selected several indicators that may affect the yield gap and determined the importance of these factors. Therefore, we summarized the variation mechanism of the grain yield gap over the past three decades and examined approaches to identify where we can close the YGAP in the future.

#### 2.3.2. Estimating the *YP*

The *YP* represents the maximum yield value achievable of the best cultivar when grown with optimal agronomy and without manageable biotic and abiotic stresses under natural resource and cropping system conditions in the specific area [28]. Methods for estimating the *YP* are crucial to *YGAP* research [39,40,78]. Generally, there are three methods for *YP* estimation, including field experiments, maximum farmer yield determination, and crop model simulation [39,57]. The former two methods are conceptually and operationally simple but exhibit notable experimental data requirements, high experimental costs, and high time costs. The latter method can consider more scenarios and treatments, but precisely quantifying all management measures in actual production is difficult to achieve [59,79,80,81]. Comprehensively, the *YP* for rice and corn was estimated by step revision model under the restrictions of light, temperature, water, and soil [82,83,84,85,86], which is referenced to the Agro-Ecological Zones modeling framework (AEZ) [87,88].

Considering the double cropping system is the main farming system in grain production in Hunan Province (Figure 1b and Figure 2a) as well that the early-season rice, late-season rice, spring corn, and autumn corn are planted between April and October. Hence, the grain potential yields (i.e., rice, corn) were estimated during their growing period (May to October) [82,83,84,85,86]. Equations (1)–(4) were adopted to calculate the photosynthetic production potential (*YQ*), light-temperature production potential (*YT*), climatic production potential (*YW*), and soil production potential (*YS*) in each cultivated land pixel, respectively.

(1) $$YQ=\frac{1\times 10^{5}}{C}\times F\times Q\times E$$

(2) $$YT=\frac{T}{30}\times YQ$$

(3) YW=f(w)×YT

(4) YS=f(s)×YW

According to previous studies [82,83,84,85,86], *C* is the calorific value of the dry matter in Equation (1), which is set to 4.25 kcal g^−1^ [82,83,84,85,86,89]. *F* is the utilization rate of light energy, with a value of 3% [82,83,84,85,86,90]. *Q* is the total solar radiation in units of kcal cm^−2^. *E* is the crop economic coefficient, and its value is generally between 0.35 and 0.5 for most grain crops, such as wheat, rice, and corn [82,83,84,85,86]. Given that rice and corn are the main grain crops in Hunan Province and that the planting area of rice exceeds 90% of the total grain planting area, a value of 0.4 is considered in this study. *T* in Equation (2) is the average temperature. The *f*(*w*) in Equation (3) is the water correction coefficient. Because there is sufficient rainfall in Hunan Province, the rainfall exceeds the amount of evapotranspiration; thus, *f*(*w*) is assigned a value of 1 in this study. In Equation (4), *f*(*s*) is the soil correction coefficient, and we employed the shared data as calculated based on soil properties, including the elevation, pH, fertility, slope, and soil texture [86].

#### 2.3.3. Calculating *YGAP* and *YGC*

In this study, *YGAP* represents the difference between potential yield (*YP*) and actual farmer yield (*AFM*), and it can effectively reflect the future grain production improvement capacity [28,38]. Equations (5) and (6) were adopted to characterize the *YGAP* in productivity per hectare (t ha^−1^) and the relative yield gap (*RYGAP*) at the county level, respectively. Moreover, considering that the variations in the yield gap correspond to the external factors changing over time and space, we calculated the *YGC* (Equation (7)) in each county and then used it to identify the determinants of *YGC* in the later analysis.

(5) $$YGAP=\bar{YS}-\bar{YFARM}$$

(6) $$RYGAP=\frac{YGAP}{\bar{YS}}\times 100\%$$

(7) $$YGC=YGAP_{i,t+1}-YGAP_{i,t}$$

where $\bar{YS}$ and $\bar{YFARM}$ in Equation (5) are the average values of the potential grain yield and actual farmer yield, respectively. *RYGAP* in Equation (6) is the relative yield gap. $YGAP_{i,t}$ and $YGAP_{i,t+1}$ in Equation (7) are the yield gaps at the beginning and end in unit *i*, respectively, of the period. A positive value of *YGC* indicates that the yield gap is increasing; in contrast, a negative value demonstrates that the yield gap is closing. It is important to note that all the above indicators are county-level statistics.

#### 2.3.4. Exploring Spatiotemporal Variations of *YGAP*

To explore the basic features of the *YGAP* dynamics over time and space, the methods of spatial statistics and spatial autocorrelation were adopted in this study. In particular, spatial autocorrelation analysis can reflect the spatial correlation characteristics via the index [37]. We first conducted a hot spot analysis to identify whether *YGAP* variations were clustered or dispersed based on their location. In addition, we recognize that the state of geographical events may be closely related to the state of the *YGAP* variation during historical periods; that is, *YGC* has a space and time lag effect. Thus, the bivariate spatial correlation method [91,92,93], which is typically considered to be the correlation between one variable and the spatial lag of another variable, was adopted to explore the relationship between the *YGCs* during different periods.

#### 2.3.5. Investigating Determinants of *YGC*

The *YGAP* is determined not only by natural conditions but also by human investments [1,26,28]. That is, climatic factors, land quality, tillage, sowing, fertilization, irrigation, and field management all affect yield gap changes [69,73,74,75,76]. In general, the main factors influencing the potential yield are natural factors, including terrain, soil, climate (e.g., solar radiation, temperature, rainfall, and CO_2_ in the environment), and genetic crop characteristics [1]. In contrast, the factors largely impacting the actual farm yield are socioeconomic factors, human investments, and market influences, such as agricultural labor, cropping systems, tillage methods, seed quality, fertilization, irrigation, drainage, and local policies [37,94,95,96]. These are the direct factors influencing the crop *YGAP*. Hence, it is crucial to understand which factors determine the yield gap change (*YGC*) to further close the *YGAP* in the future.

The spatial variations in *YGAP* are significantly correlated with changes in these factors over time and space [26]. Hence, in this study, we adopt the *YGC* as the dependent variable and choose 27 factors, including climate, topographic, socioeconomic, and human investment factors (Table 1), as independent variables to determine the main influencing factors of the *YGAP*. It should be noted that the mean values of all variables are statistically significant at the county level.

To better explore the determinants associated with the spatial variation in the *YGC*, we conducted random forest analysis, a machine learning method that has been widely used to examine the importance of influencing factors and screen the independent variables in modeling studies [77,97]. In this study, it was adopted to detect the relative importance of each impact factor (Table 1) in explaining the *YGC* at the county level. Considering the idea that the *YGC* process is dynamic, drivers should also be considered according to their inherent temporal and spatial dynamics. Consequently, we could identify invalid and dominant factors. Additionally, given that data on certain counties are absent, and agriculture is not the main industry in urban areas, the abovementioned places were eliminated. Finally, ninety-five counties were selected to analyze the influencing factors of the *YGC*. It is worth noting that we employed scores to characterize the importance, and the score value ranged from 0 to 1. The higher the value, the more important the factor is. Moreover, we considered the top ten factors in terms of their importance as the key factors and calculated the percentage according to the category and number of these factors to reflect their contribution to *YPC*. The higher the percentage is, the greater the influence on the *YGC*.

## 3. Results

### 3.1. Recent YGAP Trends in Hunan Province

#### 3.1.1. Spatiotemporal Pattern of *YGAP*

**Figure 4:**
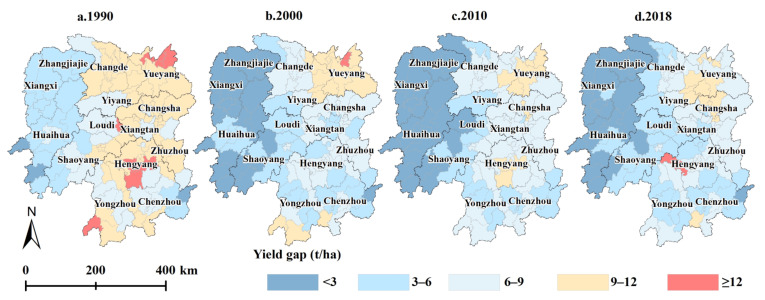
Spatiotemporal variation of *YGAP* in Hunan Province from 1990 to 2018. (**a**–**d**) refer to the yield gaps of each county in 1990, 2000, 2010, and 2018, respectively.

From 1990 to 2018, the *YGAP* in Hunan Province continued to narrow, with the average value changing from 8.57 to 5.84 t ha^−1^, a decrease of approximately 31.86%. Specifically, the minimum *YGAP* value did not significantly change after 2000, but the maximum value continued to rise. The maximum value mainly occurred in certain economically well-developed areas no longer producing grain crops, such as Furong District in Changsha city. In 2018, among the 122 counties, there were 25 counties with *YGAP* values lower than 3 t ha^−1^, largely in western Hunan. Thirty-eight counties attained *YGAP* values between 3 and 6 t ha^−1^, mainly in central and southern Hunan. Forty-three counties exhibited *YGAP* values between 6 and 9 t ha^−1^, mostly in northern Hunan. Two counties achieved *YGAP* values over 12 t ha^−1^ (Table 2 and Figure 4).

#### 3.1.2. Spatially Heterogeneity of *YGC*

**Figure 5:**
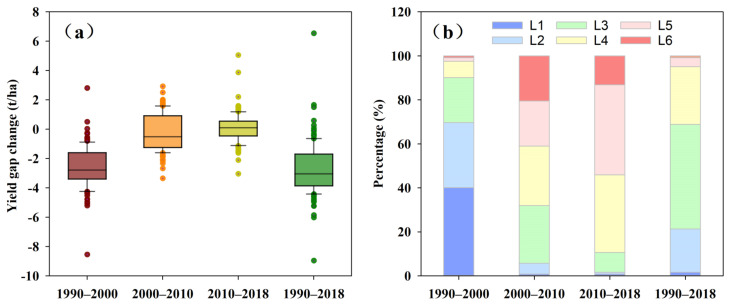
Statistical information on the *YGC* in Hunan Province from 1990 to 2018. (**a**) shows basic information on the *YGC* during the different periods, and (**b**) shows the proportion of the number of counties based on the obtained *YGC* values. In (**b**), during the periods from 1990–2000, 2000–2010, and 2010–2018, the *YGC* values were classified as L1 (<−3), L2 (−3 to −2), L3 (−2 to −1), L4 (−1 to 0), L5 (0–1), and L6 (≥1). From 1990 to 2018, the *YGC* values were classified as L1 (<−6), L2 (−6 to −4), L3 (−4 to −2), L4 (−2 to 0), L5 (0–2), and L6 (≥2).

**Figure 6:**
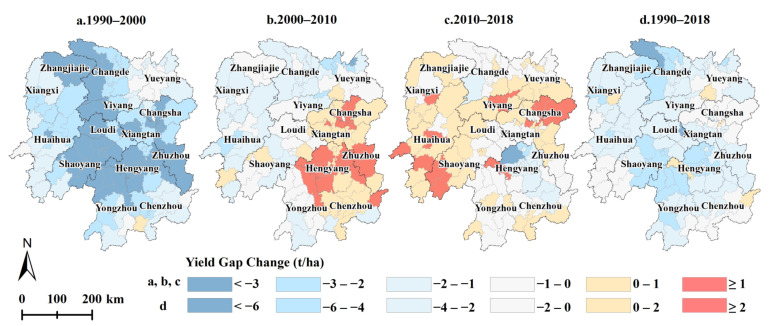
Spatial variation in the *YGCs* in Hunan Province during the different periods. (**a**–**d**) refer to the yield gap changes of each county during the periods of 1990–2000, 2000–2010, 2010–2018, and 1990–2018, respectively.

Figure 5 and Figure 6 represent that *YGAP* narrowing mainly occurred during the 1990–2000 period, and the *YGAPs* in 119 counties closed in Hunan Province. The regions where the *YGAP* narrowed within 2 t ha^−1^ were largely distributed in Yueyang, Chenzhou, Huaihua, and Xiangxi (Figure 6a), while the regions with a *YGC* value larger than 2 t ha^−1^ were mostly in Shaoyang, Hengyang, and Zhuzhou. However, from 2000 to 2010, the yield gap widened in 50 counties, mainly located in eastern Hunan (Changsha, Xiangtan, Zhuzhou) and southern Hunan (Hengyang, Chenzhou). This trend continued to expand, with the *YGAP* widening in 66 counties from 2010 to 2018, largely located in Changsha, Yueyang, Yiyang, Shaoyang, and Huaihua. However, the overall trends indicated that the *YGAP* narrowed from 1990 to 2018, and the *YGAPs* narrowed in 116 counties. Specifically, the *YGAPs* in 26 counties narrowed by more than 4 t ha^−1^, that in 58 counties, it narrowed to 2–4 t ha^−1^, and in 32 counties, it narrowed within 2 t ha^−1^.

### 3.2. Agglomeration of YGAP and YGC

#### 3.2.1. Clustering Pattern of the *YGAP* and *YGC*

**Figure 7:**
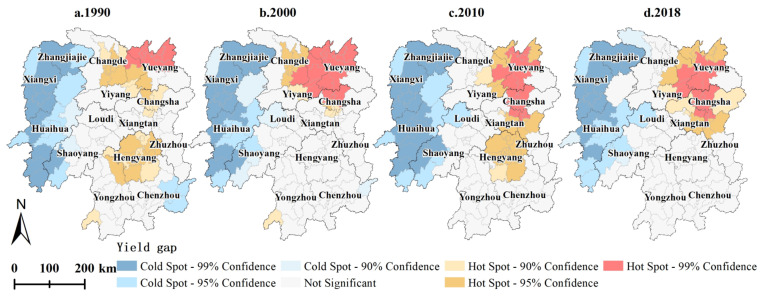
Spatial agglomeration characteristics of *YGAPs* in Hunan Province from 1990 to 2018. (**a**–**d**) are the spatial cluster maps of the *YGAPs* in 1990, 2000, 2010 and 2018, respectively.

**Figure 8:**
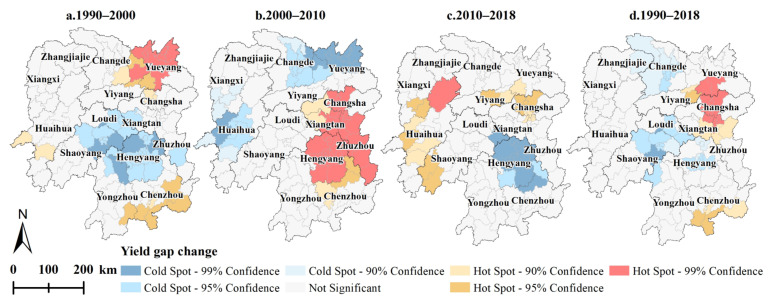
Spatial agglomeration characteristics of the *YGC* in Hunan Province from 1990 to 2018. (**a**–**d**) are the spatial cluster maps of the *YGCs* during the periods of 1990–2000, 2000–2010, 2010–2018 and 1990–2018, respectively.

The hot spot analysis results demonstrated that *YGAP* spatial clustering characteristics were evident in each year, but the changes were highly spatially divergent over time. Specifically, the cold spot areas of *YGAP* were mainly located in western Hunan (Huaihua, Zhangjiajie, and Xiangxi) each year. The hot spot areas of *YGAP* largely occurred in Changde, Yueyang, Yiyang, Changsha, and Hengyang (Figure 7). From 1990 to 2000, the cold spot areas of the *YGC* were mostly located in Shaoyang, Hengyang, and Loudi, while the hot spot areas of the *YGC* were primarily situated in Yueyang and Chenzhou (Figure 8a). From 2000 to 2010, cold spot areas of the *YGC* were observed in Yueyang, Changde, and Huaihua, and hot spot areas of the *YGC* occurred in Changsha, Xiangtan, Zhuzhou, and Hengyang (Figure 8b). From 2010 to 2018, cold spot areas of the *YGC* were located in Hengyang, whereas hot spot spatial clustering was not obvious (Figure 8c). Overall, from 1990 to 2018, cold spot areas of the *YGC* were located in the Changsha-Zhuzhou-Xiangtan city agglomeration, and cold spot areas of the *YGC* were observed in Changde, Loudi, and Shaoyang (Figure 8d).

#### 3.2.2. Spatial Autocorrelation of the *YGC* during the Different Periods

**Figure 9:**
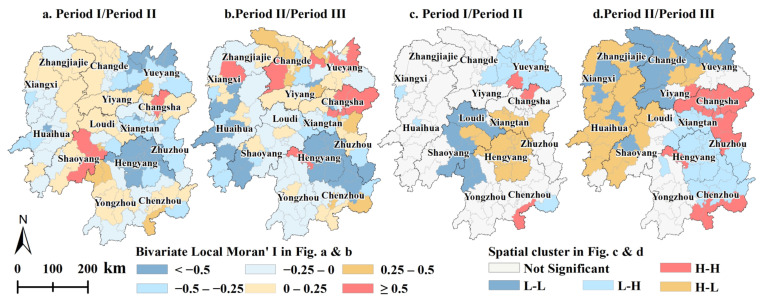
Spatial variation in bivariate local Moran’s I values of the *YGC* (**a**,**b**) and the corresponding local indicators of spatial association (LISA) cluster map of the *YGC* (**c**,**d**). Period I refers to 1990–2000, period II refers to 2000–2010, and period III refers to 2010–2018.

The value of bivariate global Moran’s I reached −0.152 between periods I and II, and the value of bivariate global Moran’s I was −0.137 between periods II and III, indicating that the *YGC* attained a weak negative spatial autocorrelation over time. Spatially, the negative values were concentrated in Hengyang, Zhuzhou, Huaihua, Xiangxi, Yueyang, and Changde during the first two periods, whilst concentrated in most counties of western Hunan, southern Hunan, and western Hunan during the last two periods. The positive values were concentrated in Shaoyang, Zhangjiajie, Yiyang, and Changsha during the first two periods and concentrated in Changsha, Yueyang, and Changde during the last two periods (Figure 9a,b).

There were high-high clusters near Changsha city and in southern Chenzhou, indicating that the yield gaps in these locations and surrounding neighborhoods continued to widen (Figure 9c,d). Low-low clusters occurred in Loudi and Shaoyang during the first two periods and around Changde, Yiyang, and Yueyang during the last two periods, which indicates that the yield gaps in these regions were significantly narrowing (Figure 9c,d).

### 3.3. Determinants of the YGC

**Figure 10:**
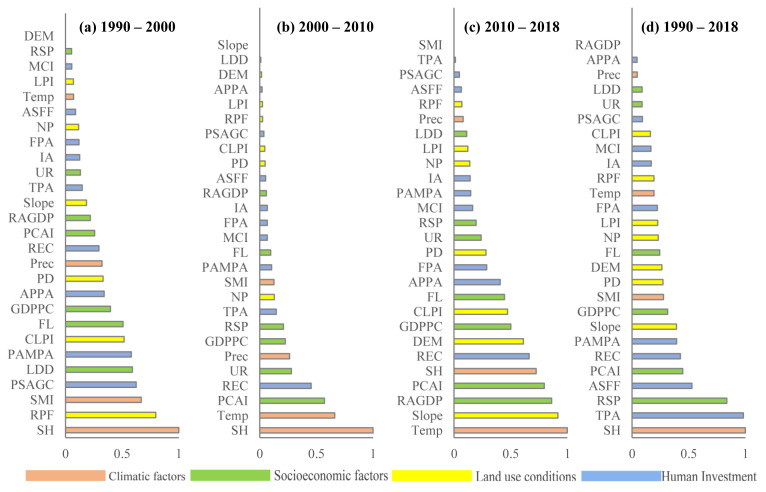
Importance of the influence factors for *YGC* during the different periods. (**a**–**d**) are the relatively scores of each variable to yield gap change during the periods of 1990–2000, 2000–2010, 2010–2018 and 1990–2018, respectively.

Normalized importance scores of the influencing factors are shown in Figure 10. We counted the frequency of the top ten scoring factors during each period, and we found that the most frequent factors included *GDP* per capita (*GDPPC*, four times) and sunshine hours (*SH*, four times), followed by the per capita annual net income of farmers (*PCAI,* three times) and rural electricity consumption (*REC*, three times), and finally cultivated land quality level (*CLPI*), farm labor (*FL*), power of agricultural machinery per area (*PAMPA*), rural household population (*RSP*), slope, solar mediation intensity (*SMI*), temperature (*Temp*), and tractor-plowed area (*TPA*), with a frequency of two. To a certain extent, this reflected that the *YGC* was greatly influenced by these factors.

**Figure 11:**
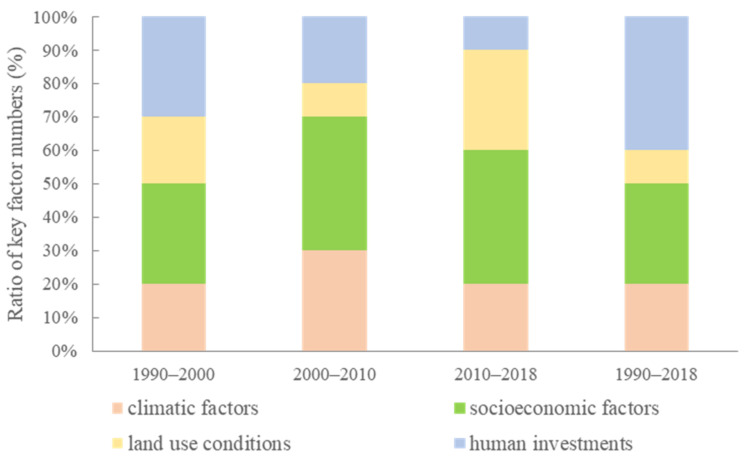
Contribution of the key factors influencing the *YGC* between 1990 and 2018.

The importance of these factors revealed distinct characteristics during the different periods (Figure 10). From a temporal perspective, during the former period, climatic factors and land use conditions were the main factors influencing the *YGC*, but during the latter period, the *YGC* was mainly determined by climatic and socioeconomic factors. Throughout the whole study period, socioeconomic factors and human investment variables were relatively important to the *YGC*. From the perspective of the variable types, among the climatic factors, sunshine hours (*SH*) and temperature (*TEMP*) remained the main influencing factors of the *YGC*, while the impacts of solar mediation intensity (*SMI*) and precipitation (*PREC*) on the yield gap were relatively limited. Among the socioeconomic factors, *GDP* per capita (*GDPPC*), the per capita annual net income of farmers (*PCAI*), and farm labor (*FL*) were comparatively important. Among the land use conditions, the area ratio of paddy fields (*RPF*), slope, and patch density (*PD*) were relatively important. Among the human investment variables, rural electricity consumption (*REC*), the proportion of the area sown with grain crops (*PSAGC*), and the tractor-plowed area (*TPA*) were comparatively important (Figure 10). As shown in Figure 11, human investment variables and socioeconomic factors were the major influencing factors of the *YGC* from 1990 to 2018, followed by climatic factors and land use conditions. The importance of human investment variables decreased while the importance of socioeconomic factors increased. Overall, socioeconomic factors are the dominant determinants of the *YGC*, especially after 2000, and land use conditions yield relative importance for the *YGC*.

## 4. Discussion

According to the existing studies [82,83,84,85,86], the *YP* is based on a step-by-step revision model involving light, temperature, water, and soil data (Equations (1)–(4)). The accuracy of the estimation method was verified by comparing it with the actual output of the experimental field plot in Taoyuan County, Changde city, Hunan Province [82]. Some scholars further corrected the climate production potential by using soil properties to obtain the *Y_s_* [86,98,99]. In recent years, this method has been widely used in studies associated with grain production potential measurement and cultivated land protection in China [72,85,86], implying that the method for estimating potential yield is reliable. Our estimated mean values of *YS* were approximately 13.53 t ha^−1^ in 1990 and 11.90 t ha^−1^ in 2018 in Hunan Province. Compared with other studies, which showed that the average *YS* of grain crops in double-cropping systems is between 10 t ha^−1^ and 14 t ha^−1^ in Hunan Province by using the ESAP model [78] and the GAEZ model [20,71], indicating that our method for estimating potential yield is robust. Second, considering the cropping system is crucial in estimating the *YGAP* [75,100,101], and double cropping is the main farming system in grain production in Hunan Province (Figure 1b and Figure 2a) [71]. In this study, the estimations of potential yield are under a double-cropping system with rice in paddy fields and corn in drylands. Third, we use the statistical data at the county level as the actual farm yields based on the following considerations. On the one hand, the county is the basic and an important unit of government management in China, and the statistical data can reflect the average level of actual grain yields. This method is consistent with the GYGA website (http://www.Yieldgap.org, accessed on 23 September 2021), which is widely used in national *YGAP* analysis [20]. On the other hand, despite an increasing number of studies employing remote sensing technology and crop growth models to estimate the actual yields in recent years, it is difficult to obtain high-quality data in 1990 and 2000 to perform this work. Comprehensively, the estimation method for the *YGAP* in this study is simple and conducive to generalization.

**Figure 12:**
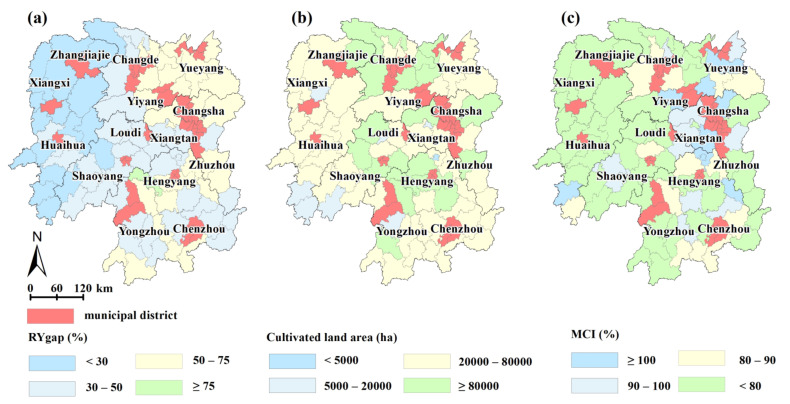
Capacity for potential yield (**a**), cultivated land area (**b**) exploitation, and multiple cropping index of grain crops (**c**) in 2018.

As we know, total grain outputs are closely related to the harvested area and yield per unit area. Existing literature showed that both of them have a larger gap between potential and actual activities in China, especially in the regions such as the middle and lower reaches of the Yangtze River [19,20,102]. Thus, there are two ways to increase food production without cropland expansion. The first one is to improve the cropping intensity [19,103,104,105], another one is to close the yield gap [12]. We found that the yield gap in Hunan Province was 5.84 t ha^−1^ (49.07% of the potential yield), and the multiple cropping index of grain crops was 0.8 in 2018, indicating that there is a high grain production potential to exploit. This result is consistent with Ye et al. [103], which showed that the arable land intensity of Hunan Province is relatively low. However, this does not mean that all counties in Hunan Province exhibit a high potential. Studies have demonstrated that the attainable yield ranges from approximately 75% to 85% of the potential yield; that is, approximately 15% to 25% of the yield gap cannot be exploited [20,38,39]. Consequently, we should focus on areas where the yield gap is greater than 25% when identifying regions for grain production enhancement. Here we chose a value of 30%. That is, without considering regional planning, socioeconomic, and other factors, 98 counties were identified as important areas where grain production enhancement could be achieved, 34 of which are municipal districts and should be excluded, which suggests that there are 64 counties with much capacity for yield improvement (Figure 12a).

Our results also indicate that the regions located in northern, central, and southern Hunan exhibit great grain production improvement potential by integrating the yield gap (Figure 12a), cultivated land area (Figure 12b), and multiple cropping index of grain crops in 2018 (Figure 12c). These regions possess good natural conditions, the terrain is relatively flat, and it is more suitable to perform large-scale agricultural activities within the context of severe rural labor migration. However, a previous study found that supplementary cultivated land is mainly distributed in northern, southern, and western Hunan [72], indicating that there is an inconsistency in the spatial distributions of supplementary cultivated land and the *YGAP*, which may result in the low economic efficiency of projects. Other studies reported that the yield gap for early-season rice in Hunan Province is generally higher than that for late-season rice and that the yield gaps are the largest in the northern region for both early- and late-season rice [106]. Consistent with this result, our study also demonstrated that there is much capacity for yield enhancement in northern Hunan, followed by eastern Hunan, southern Hunan, and central Hunan, while western Hunan contains less capacity for yield improvement. Comprehensively, during the implementation of cultivated land protection policies (e.g., the cultivated land balance policy and high-standard farmland construction), these areas should be prioritized to close the yield gap. According to the sown area of grain crops in these 64 counties in 2018, an additional 1.11 million tons of grain can be produced if the actual grain yields can be increased by 5%, which is equivalent to 3.71% of the total grain output in 2018.

As we mentioned before, the yield gap is determined by a combination of natural and socioeconomic conditions, and notable spatial and temporal heterogeneity occurs in the main influencing factors and mechanisms [1,28,38,39]. However, in addition to farm household field management, the macroeconomic environment also has an important impact on the *YGC* [51,69,76,107]. For example, with rapid urbanization, the economic efficiency of agriculture is low, people prefer to plant other crops to obtain higher economic returns, and rural labor migration and rural aging further result in the abandonment of cultivated land [108]. Therefore, we analyzed the evolution mechanism of the *YGC* at the county scale, considering not only natural factors but also socioeconomic factors, which are of great significance to guide policy-making related to grain production under real socioeconomic environments.

Previous studies have reported that the potential yield is positively correlated with the total solar radiation, a decrease in radiation leads to a decrease in the potential crop yield, and the potential yield is negatively correlated with the temperature within a certain range [76]. According to the calculation method of the yield gap (Equations (1)–(5)), all the climatic factors attained a positive relationship with the potential yield. However, we found that not all climatic factors played an important role in the *YGC*. Among these factors, sunshine hours (*SH*) and temperature (*Temp*) were relatively more important than other factors in Hunan Province because *SH* can directly determine the duration of photosynthesis, and temperature can influence the cropping system. This is similar to the results of many other studies [1,109,110]. Deng et al. revealed that the increase in temperature should be synchronized with the increase in water resources [20]. Otherwise, drought may occur, causing a reduction in yield, implying that food production can also be increased by improving irrigation conditions and implementing land consolidation projects of dryland to paddy in places with abundant solar resources and low irrigation ratios, such as southern, central Hunan.

Among the socioeconomic factors, land use conditions, and human investment variables, it seems that field management and human investment variables, such as tractor-plowed area (*TPA*), area of soil testing and formula fertilization (*ASFF*), rural electricity consumption (*REC*), and power of agricultural machinery per area (*PAMPA*), exert a large impact on the yield gap, but the impact decreases over time. Especially from 2010 to 2018, the yield gap was mainly influenced by macroscopic socioeconomic variables such as the ratio of agricultural *GDP* (*RAGDP*) and the per capita annual net income of farmers (*PCAI*); it appears that with economic development, more and more farmers are unwilling to engage in food production activities [76]. Moreover, during this period, changes in slope imposed a significant effect on the yield gap. According to the data, the average slope value changed from 6.20 to 6.22, which may be caused by the implementation of the cultivated land balance. Specifically, the occurrence of occupied cultivated land is mainly in flat areas, while the slope of supplementary land is relatively large. Additionally, Sun et al. calculated that the correction coefficient of soil is about 0.42 in Hunan Province [86], implying that there is great potential for increasing grain production by improving the cultivated land quality.

Overall, some shortages remain in this study, and further work is needed. Firstly, the potential yield is the ideal yield and is almost impossible to achieve, and the exploitable yield potential is more instructive for agricultural production. However, due to the absence of historical data, it is difficult to estimate the exploitable yield potential for each year. Hence, we calculated the land potential productivity with the step-by-step light, temperature, water, and soil revision model instead. Indeed, the value of the estimated potential yield is relatively larger than that of the available yield, but this does not affect the overall distribution pattern of the yield gap. Secondly, other studies have adopted remote sensing to estimate actual farm yield values [66], which may be more accurate than the use of statistical data. Thirdly, we analyzed the importance of each factor rather than exploring their spatiotemporal influence mechanisms on the *YGC*, which may limit the applicability of our results on a spatial scale.

Despite these limitations, the systematic methodological framework developed in this study provides a new perspective aimed at coupling the yield gap, determinants, and land use. Related analysis methods could be applied to support cultivated land utilization. In the future, to improve the applicability of the research results, further optimization of the estimation method of the exploitable yield potential and the actual farm yield is needed. In addition, considering only the yield gap to identify regions is insufficient. For instance, certain areas exhibit an irreversible trend of nonagricultural production and nongrain planting due to urbanization, and the yield gap in these regions is inevitably gradually expanding (e.g., Furong district), but these regions cannot be considered for grain production enhancement. Therefore, it is necessary to integrate land resources, population migration, and regional economic development trends under different scenarios to comprehensively identify regions where grain production could be improved and develop a plan for sustainable cultivated land use, agricultural investment, etc. [111,112].

## 5. Conclusions

In this study, we established a framework applicable to *YGAP* research and preliminarily implemented the framework. Specifically, we employed a step-by-step revision model involving light, temperature, water, and soil data to assess the potential yield, obtained the actual farm yield based on socioeconomic statistics, and then adopted spatial analysis and spatial statistics methods to evaluate the spatiotemporal evolution and clustering characteristics of the *YGAP* at the county level in Hunan Province, which is a major grain production region. Finally, the random forest model was adopted to investigate the key influencing factors of the *YGC*. Based on these analyses, we determined the possible grain yield improvement capacity in Hunan. The proposed framework for *YGAP* research exhibits good application prospects, and these application schemes could optimize the interaction between natural conditions, the social environment, and management practices. Once popularized, these applications could provide the potential to enhance the allocation of funds for farmland consolidation and could increase the grain yield.

Our results revealed that the *YGAP* in Hunan Province continued to narrow from 1990 to 2018, and the average value changed from 8.57 t ha^−1^ (63.36% of the potential yield in 1990) to 5.84 t ha^−1^ (49.07% of the potential yield in 2018), indicating that there exists a high grain production potential to exploit. From the perspective of dynamics, there is large spatial heterogeneity in the variation of *YGCs*. Specifically, the *YGAPs* in 116 counties have narrowed. Of which, 26 counties narrowed by more than 4 t ha^−1^, 58 counties narrowed from 2–4 t ha^−1^, and 32 counties narrowed within 2 t ha^−1^.

Additionally, Our results found that during the former period, climatic factors and land use conditions were the main factors influencing the *YGC*, but during the latter period, the *YGC* was mainly determined by climatic and socioeconomic factors. Overall, the *GDP* per capita (*GDPPC*), sunshine hours (*SH*), per capita annual net income of farmers (*PCAI*), and rural electricity consumption (*REC*) play a key role in *YGCs*, reflecting that socioeconomic factors are becoming increasingly important for grain production.

Notably, *YGAP* analysis can identify the corresponding distribution for yield improvement purposes. However, considering only the *YGAP* to identify regions is insufficient because agricultural production is also influenced by other factors. Integrating the trends of land use, population migration, and regional development strategies when formulating policies related to grain production and agricultural investment is needed. Therefore, considering the yield gap, cultivated land resources, multiple cropping index, and development orientation, the 64 identified counties, which are mainly located in northern, central, and southern Hunan and have a yield gap greater than 30%, constitute the major areas for grain production enhancement. Our findings offer important scientific value to better understand the law and mechanism of the *YGC* in Hunan Province and support the decision-making process involving cultivated land use.

## Figures and Tables

**Table 1:** Description of the variables.

Variable Category	Variables	Units	Data Sources
Dependent variable	Yield gap change (*YGC*)	tons per hectare	Calculated with Equation (7)
Climatic factors	Sunshine hours (SH)	hours	China Meteorological Data Service Centre (http://data.cma.cn, accessed on 13 February 2021) and National Tibetan Plateau Data Center (http://data.tpdc.ac.cn, accessed on 13 February 2021)
	Solar mediation intensity (SMI)	watt per kilometers	
	Temperature (Temp)	°C	
	Precipitation (Prec)	mm	
Socioeconomic factors	Rural household population (RSP)	ten thousand people	Hunan Provincial Bureau of Statistics
	Land development degree (LDD)	%	Resource and Environmental Science Data Center (RESDC)
	Population urbanization rate (UR)	%	Hunan Provincial Bureau of Statistics
	Farm labor (FL)	ten thousand people	Hunan Provincial Bureau of Statistics
	Gross domestic product (GDP) per capita (GDPPC)	CNY per capita	Hunan Provincial Bureau of Statistics
	Ratio of the agricultural GDP (RAGDP)	%	Hunan Provincial Bureau of Statistics
	Per capita annual net income of farmers (PCAI)	RMB per capita	Hunan Provincial Bureau of Statistics
Land use conditions	Elevation of cultivated land (DEM)	m	Advanced Land Observing Satellite-1 (ALOS), Japan Aerospace Exploration Agency
	Slope of cultivated land (Slope)	degree	ALOS, Japan Aerospace Exploration Agency
	Area ratio of paddy fields (RPF)	%	Chinese Academy of Sciences
	Number of patches (NP)	–	Chinese Academy of Sciences
	Patch density (PD)	number per hectare	Chinese Academy of Sciences
	Largest patch index (LPI)	–	Chinese Academy of Sciences
	Cultivated land quality level (CLPL)	level	Department of Natural Resources of Hunan
Human investment	Proportion of the sown area of grain crops (PSAGC)	%	Hunan Provincial Bureau of Statistics
	Multiple cropping index of grain crops (MCI)	%	Hunan Provincial Bureau of Statistics
	Agricultural practitioners per area (APPA)	person per hectare	Hunan Provincial Bureau of Statistics
	Rural electricity consumption (REC)	ten thousand watt	Hunan Provincial Bureau of Statistics
	Amount of fertilizer per area (FPA)	tons per hectare	Hunan Provincial Bureau of Statistics
	Tractor-plowed area (TPA)	ha	Hunan Provincial Bureau of Statistics
	Irrigated area (IA)	ha	Hunan Provincial Bureau of Statistics
	Power of agricultural machinery per area (PAMPA)	kilowatt per hectare	Hunan Provincial Bureau of Statistics
	Area of soil testing and formula fertilization (ASFF)	hectare	Hunan Provincial Bureau of Statistics

Note: all indicators are obtained or calculated from the corresponding data of 1990, 2000, 2010, and 2018.

**Table 2:** Statistical information on YGAP in Hunan Province from 1990 to 2018.

Year	*YGAP* (t ha^−1^)			<3 t ha^−1^		3–6 t ha^−1^		6–9 t ha^−1^		9–12 t ha^−1^		≥12 t ha^−1^	
	Maximum	Minimum	Mean	Number	Ratio	Number	Ratio	Number	Ratio	Number	Ratio	Number	Ratio
1990	14.82	1.63	8.57	3	2.46%	25	20.49%	28	22.95%	58	47.54%	8	6.56%
2000	13.54	0.11	5.95	23	18.85%	37	30.33%	43	35.25%	16	13.11%	3	2.46%
2010	15.06	0.24	5.74	31	25.41%	21	17.21%	56	45.90%	13	10.66%	1	0.82%
2018	17.28	0.15	5.84	25	20.49%	38	31.14%	43	35.25%	14	11.48%	2	1.64%

## Data Availability

Data is contained within the article.
